# Gender Differences in Maladaptive Cognitive Schema in Orphans in Dakahlia, Egypt

**DOI:** 10.1155/2013/373120

**Published:** 2013-12-24

**Authors:** Abdel-Hady El-Gilany, Mona A. El-Bilsha, Azza Ibrahim

**Affiliations:** ^1^Faculty of Medicine, Mansoura University, Mansoura 35516, Egypt; ^2^Faculty of Nursing, Mansoura University, Mansoura 35516, Egypt

## Abstract

The objective of this study was to assess the gender differences of maladaptive cognitive schema among orphans in Dakahlia governorate orphanages. A cross-sectional comparative study included 152 orphan boys and 48 orphan girls in all orphanages homes in Dakahlia governorate, Egypt. Data collection tools included a structured interview questionnaire for personal data; early maladaptive schema questionnaire-short form (EMSQ-SF). The mean score of the total YSQ and all the subscales, except self-sacrifice and unrelenting standards, are significantly higher among females than males. Attention should be given to the psychological care of the orphans especially security, trust, confidence, and autonomy with more attention to orphan girls.

## 1. Introduction

Institutionalized children are at increased risk for developing emotional and behavioral problems for a variety of reasons. Among these, death of parents makes children vulnerable and predisposes them to physical and psychological risks [1].

Early maladaptive schemas (EMS) are defined as deep cognitive structures constituted by beliefs about self, others, and the environment. Such maladaptive schemas distort information about the relation between the subject and the environment, which create negative automatic thoughts and depression [2].

Schema theory is one possible way of explaining the relationship between experiences in childhood, inborn temperament, and psychosocial or person-logical outcomes in adulthood. This theory proposed negative childhood experiences (NCE) as a central feature in the development of early maladaptive schema (EMS) [3]. According to Young and colleagues, NCE interfere with normal development by interrupting the fulfillment of core emotional needs. Interruption of a child's sense of safety, nurturing, or belonging can contribute to the formation of EMS, which subsequently influence an individual's way of perceiving and experiencing the world and/or internal processes such as emotions, thoughts, and memories [4].

Gender-associated perception, cognition, and behavior result from lifelong social learning acting upon seemingly innate neuropsychological and psychobiological adaptations [5]. We proposed that girls are more likely to experience maladaptive schema than orphan boys.

In Egypt, orphanage harbors either true orphans or foundlings abandoned by mothers after illegal pregnancies. There is a lack of research that highlights the psychological adjustment of orphans in general and gender differences in cognitive schema. So the present study was to assess the gender differences of maladaptive cognitive schema among orphans in Dakahlia governorate orphanages, Egypt.

## 2. Population and Methods

This is a cross-sectional comparative study carried out on all children in 10 orphanages in Dakahlia governorate, Egypt, during the period from June to December 2011. The orphanages are El-Baneen, Dar-Ebnaty, El-Banat, Marmena Dar El-Nahda, and El-kalema El-kebtia in Mansoura city, Tahseen-El-Seha and El-Safa in Talkha city, Fagr-El-Eslam in Bilqas city, and El-ketab-El-moqadas and El-Amal in Mit Ghamer city.

Inclusion criteria were orphans in the age of 6 to 18 years of both sexes and no mental retardation. There were 262 orphans resident in the above mentioned orphanages. Two hundred orphans were included in the study. Forty-two were excluded because of their age (either below 6 or above 18 years) and 20 involved in the pilot were excluded from the full-scale study. 

The study protocol was approved by the Research Ethics Committee of the Faculty of Nursing, Mansoura University. The nature of the study is harmless; all data are kept confidential and used only for the research purpose. The study subjects willingly agreed to participate in the study and gave their verbal consent, and each participant was free to withdraw at any time throughout the study. Before the interview, children were informed about the purpose of the study and were assured about confidentiality of data. The interview took about 20–30 minutes. Sometime we needed more than one session to complete the data especially with children less than 12 years.

A pilot study was carried out on 20 children in three orphanages in Dakahlia governorate. These orphanages were purposively selected because the number of resident children was less than 10 in each home and they were excluded from the full-scale study. They were chosen to test reliability, clarity, and simplicity of the tool used. The subscale of enmeshment is not applied to the respondents as all children living in the orphanage are away from their parents, so the response to its five items is constant. Following the pilot study, the final form of tool was reconstructed and made ready to use.

Structured interview questionnaire was used to collect the following.

1. Sociodemographic data: for example, age, gender, level of education, and duration of stay in orphanage.
2. Early maladaptive schema questionnaire-short form (EMSQ-SF): this scale was developed by [6]; it is designed to measure 15 primary early maladaptive schemas. The scale comprises five domains and 15 subscales each measured through 5 items. In total there are 75 items and each item is measured on a six-point scale that ranges from 1 = “Completely untrue of me” to 6 = “Describes me perfectly.” Higher scores on the YSQ-S subscales (e.g., 5 or 6) indicate that a maladaptive core belief is present. Scores are summed for each subscale, for a total subscale score, and these scores are summed for a total YSQ score. Possible scores for each subscale range from five to 30 with the total score of the YSQ-S ranging from 75 to 450.

The YSQ-SF was initially independently translated by the first two researchers; they are proficient in English with an Arabic native language. The two translations were compared and when differences were identified, the texts were modified to obtain consensus between the two translations. The consensus Arabic version was back translated into English by two translators who were unaware of the original version. The two English versions were compared with the original English version. The differences were analyzed, and questions and/or response choices were rewritten when necessary, thus proving a second Arabic version (slang language). All the misunderstood items were replaced and discrepancies were resolved by the researchers. Content-related validity and appropriateness of translation of the study tool were tested by 6 experts in the field of education from the Faculties of Nursing, Medicine, and Education, Mansoura University. The necessary modifications to the tool were done based on expert's suggestions (e.g., modifications related to Arabic translation).

The YSQ includes 75 items covering five domains with respect to

1. disconnection/rejection domain (questions from 1 to 25);
2. impaired autonomy and performance domain (questions from 26 to 45);
3. other-directedness domain (questions from 46 to 55);
4. overvigilance and inhibition domain (questions from 56 to 65);
5. impaired limits domain (questions from 66 to 75).

The subscale of enmeshment is not applicable to the respondents as all children live in the orphanage away from their parents, so the response to its five items is constant.

A number of studies have investigated the YSQ and the YSQ-S [6] and found good reliability. Using a large clinical and student sample Schmidt et al. [7] found high to very high Cronbach's alpha coefficients that ranged from 83 for the enmeshment subscale to 96 for the defectiveness subscale. Test-retest reliability over a three-week period ranged from a low 50 for dependency to a high of 0.82 for emotional deprivation.

Reliability of the Arabic version was tested during the pilot study. Cronbach's *α* of the total scale is 0.85. It ranges from 0.55 to 0.89 in different subscales. The overall score ranged from 196 to 369 with a mean of 283 ± 37.7. The intra- and interrater correlation coefficients of the total score are the same and equal 0.88. The intrarater correlation of different subscales ranges from 0.71 to 1.0 and the inter-rater correlation of different subscales ranges from 0.70 to 0.99.

Data were analyzed using (Statistical Package for Social Sciences) SPSS version 16.0. Qualitative variable was presented as number and percent. Comparison between groups was done by Chi-Square test. Quantitative variables were presented as Mean ± SD and unpaired Student's *t*-test was used for comparison between genders. *P* ≤ 0.05 was considered to be statistically significant.

## 3. Results

In Table 1, boys and girls were matched regarding their educational level, age, and duration of stay in the orphanages.

Table 2 shows that girls reported a significantly higher mean total YSQ than boys (288.3 versus 246.1; resp.). The same pattern was observed in all subscales except self-sacrifice and unrelenting standards (the mean scores of these two subscales didn't differ between females and males).

## 4. Discussion

Maladaptive schemas are presumed to develop early in life through negative interactions with primary caregivers and make people vulnerable to psychological problems when confronted with stress [8].

The total scores of YSQ are significantly higher in orphan girls than in orphan boys. Females are more emotionally unstable and exposed to stressors than males; they need more love, warmth, affection, and so forth. On the other hand, the Arabic culture enhances male socialization more than female which helps in developing health cognitive schema. A previous study documented that girls have more maladaptive cognitive schema than boys [9]. Girls reported higher feeling of emotional deprivation and abandonment than boys. Child girls are more sensitive and more fragile than boys so they need more emotional care. Similarly Lamba and Singh [10] support the idea that the child who lives in institutional care yet again experiences emotional deprivation. Our culture enhances males' independence while females may feel insecure as they may lose their caregivers at any time; this may contribute to the feeling of abandonment among females.

Another common aspect among females is shame. This result is supported by Deblinger and Runyon [11] who revealed that there are individual differences in shame responding and girls generally may be more prone to experience shame than boys. This may be due to rapid change and self-focus that occurs in the adolescence period which makes girls prone to engage in negative self-evaluations and social comparisons [12].

The present study reported that girls express greater feelings of abuse and mistrust than boys. This result may be due to lack of social power of girls that makes them more vulnerable than boys to specific major trauma particularly sexual abuse. In the present study, social isolation is more common among females. This finding contradicts Vandervoort [13] who reported that males are more socially alienated than females. This maybe due to the stigma of being an orphaned which has impact on girls and there is a little chance for the girls to get out with friends for playing, shopping, and so forth. Moreover, the rules of our culture prevent females from becoming more sociable.

Regarding autonomy and performance domain, the belief that one has failed or is inadequate relative to one's peers in areas of achievement and exaggerated fear that is imminent catastrophe will strike at any time. In the present study girls reported higher feeling of failure to achieve. It may be due to the eastern tradition that pays attention to males' achievement more than females' achievement.

This study shows that girls report greater vulnerability to harm than boys. This agrees with Satterfield et al. [14] who have found that females are more risk-averse than men. It is rationalized as the same as abuse or it may be due to reduced social and formal decision making power held by females.

We found that the mean score of self-sacrifice is higher among girls. In a similar study Calvete et al. [15] found that self-sacrifice is more significant to females; they value their relationships as key to self-worth. This could be explained as females are born with inherited sense of motherhood which is similar to self-sacrifice or may be related to acquiring positive self-esteem through helping or sacrificing for others.

The child has an excessive inhibition of spontaneous action, feeling, or communication, usually to avoid disapproval by others, feelings of shame, and criticism by others [3]. The present study revealed that girls reported greater feelings of emotional inhibition than boys. Our culture prohibits females' social expression. This result contradicts Cossette et al. [16] who stated that females have more tendencies for both verbal and nonverbal emotional expression. Moreover, Naito et al. [17] stated that females are more aware of their emotions and report more complex emotional experiences, and a greater willingness to express their emotions openly. Also, they show stronger tendencies to regulate them to adapt to changing social circumstances compared with males.

As for impaired limits domain, the current study revealed that unrelenting Standards and Entitlement Grandiosity are significantly higher among females. This result is congruent with the attitude of most of children who prefer to feel that they are the best and the first.

Based on the results of this study we recommend giving attention to the psychological care of the orphans especially security, trust, confidence, and autonomy with more attention to female orphans. Further research should be carried out on community sample of children at the national level.

## 5. Study Limitations

This study was done on children in orphanage in a single governorate, and its results do not necessarily apply to all children in the community all over Egypt. The subscale of enmeshment was excluded as it is not applied to the respondents as all children living in the orphanage are away from their parents, so the response to its five items is constant with a total subscale of 5.

## Figures and Tables

**Table 1:** Comparison between boys and girls regarding age, duration of stay in orphanage, and educational level.

	Total (200) (Mean ± SD)	Boys (152) (Mean ± SD)	Girls (48) (Mean ± SD)	Significance
Age (years)	13.9 ± 2.2	13.99 ± 2.4	13.5 ± 1.5	*t* = 1.3, *P* = 0.2
Duration of stay (years)	7.8 ± 3.2	7.9 ± 2.9	7.5 ± 4.1	*t* = 0.8, *P* = 0.4
Education (*n* & %):				*χ* ^2^ = 2.3, *P* = 0.4
Primary	64 (32.0)	45 (29.6)	19 (39.6)	
Preparatory	93 (46.5)	75 (49.3)	18 (37.5)	
Secondary	43 (21.5)	32 (21.1)	11 (22.9)	

**Table 2:** Variation of the score of maladaptive schema according to gender.

Subscales	Gender		Significance
	Boys (Mean ± SD)	Girls (Mean ± SD)	
Disconnection/rejection			
Emotional deprivation	17.15 ± 5.17	19.68 ± 4.27	*t* = 3.07, *P* ≤ 0.001
Abandonment/instability	20.79 ± 5.68	23.22 ± 5.35	*t* = 2.61, *P* ≤ 0.001
Mistrust/abuse	16.61 ± 4.92	19.06 ± 4.17	*t* = 3.10, *P* ≤ 0.001
Social isolation	15.68 ± 5.78	20.54 ± 4.90	*t* = 5.08, *P* ≤ 0.001
Defectiveness/shame	16.36 ± 5.51	20.54 ± 4.74	*t* = 4.69, *P* ≤ 0.001
Autonomy/performance			
Failure to achieve	16.66 ± 5.36	20.20 ± 4.74	*t* = 4.09, *P* ≤ 0.001
Dependence/incompetent	16.86 ± 5.78	21.60 ± 5.35	*t* = 5.03, *P* ≤ 0.001
Vulnerability to harm	16.11 ± 4.83	19.41 ± 4.52	*t* = 4.18, *P* ≤ 0.001
Enmeshment*	—	—	—
Other-directedness			
Subjugation	17.56 ± 5.61	20.56 ± 4.92	*t* = 3.3, *P* ≤ 0.001
Self-Sacrifice	18.90 ± 5.26	19.72 ± 4.33	*t* = 0.98, *P* = 0.3
Over vigilance/inhibition			
Emotional inhibition	17.24 ± 4.87	21.08 ± 4.91	*t* = 4.74, *P* ≤ 0.001
Unrelenting standards	18.55 ± 5.91	19.89 ± 5.11	*t* = 1.4, *P* = 0.2
Impaired limits			
Entitlement grandiosity	18.40 ± 5.55	21.52 ± 5.56	*t* = 3.38, *P* ≤ 0.001
Insufficient self-control	18.22 ± 5.36	20.37 ± 5.71	*t* = 2.38, *P* ≤ 0.001

Total YSQ	246.1 ± 38.6	288.3 ± 32.67	*t* = 6.82, *P* ≤ 0.001

*Not possible to calculate because this variable is constant.
